# Dental Maturation in Two Groups of Children Born in 1969–1973 and 2005–2010

**DOI:** 10.3390/dj11110248

**Published:** 2023-10-25

**Authors:** Astrid Rathcke Poulsen, Liselotte Sonnesen

**Affiliations:** Section of Orthodontics, Department of Odontology, Faculty of Health and Medical Sciences, University of Copenhagen, DK-2200 Copenhagen, Denmark; ar-poulsen@hotmail.com

**Keywords:** retrospective study, comparison, dental maturation, dental age, permanent dentition

## Abstract

This study compared differences in dental maturation and dental age in Scandinavian children born in 1969–1973 and 2005–2010. The study was based on 130 ethnic Scandinavian children, who were divided in two groups corresponding to the time periods and matched on age and sex. Each group consisted of 65 children (30 girls, 35 boys, mean age 9.29 years and 9.30 years, respectively). Dental maturation was analysed by orthopantomographs, in accordance with Demirjian and Haavikko. Dental age was analysed by orthopantomographs in accordance with Demirjian. Boys and girls were analysed separately by linear regression analysis adjusted for age. For the children born in 2005–2010, teeth matured significantly earlier (Demirjian: 0.21 maturities, CI 95% 0.11–0.31, *p* < 0.000. Haavikko: maxilla: 0.39 maturities, CI 95% 0.21–0.56, *p* < 0.000. Mandible: 0.42 maturities, CI 95% 0.17–0.67, *p* = 0.001) and the dental age was significantly higher (Demirjian: 6.04 months, CI 95% 0.23–0.77, *p* < 0.000) in comparison with the children born in 1969–1973. In conclusion, the teeth of Scandinavian children born 2005–2010 matured significantly earlier than those of children born in 1969–1973. The results may prove valuable in forensic dentistry, pedodontics, and for the timing of pedodontic and orthodontic treatment.

## 1. Introduction

Childhood puberty and skeletal maturation (SM) are occurring earlier than before [1,2,3,4,5]. The reason is multifactorial and is partly due to socio-economic growth, improved nutritional conditions, and environmental changes [1,6,7]. In addition to earlier maturation, children reach peak height velocity (PHV) earlier and have an increased growth rate when growth is at its maximum [1]. During the years 1920–1990, an increase in body height of approximately 7 cm for girls and approximately 11 cm for boys has been found [3].

As children reach puberty earlier and the skeleton matures earlier, teeth may also mature earlier. An acceleration in dental age has been found in various previous time periods [3,8,9,10,11], but this has not been examined on Scandinavian children for a recent time period. Some studies speculate that dental development is not affected by external factors but is controlled primarily by genetics [12], while other studies speculate that teeth are also affected by external factors [3,13,14]. The risk of early puberty is increased amongst children with stress, anxiety, inactivity, and a high-energy intake [5,15,16]. Advanced dental maturation (DM) has also been found in children with a high-energy intake [2,16,17]. However, delayed DM has been found in children with deficiencies in growth, tooth agenesis, and endocrine disorders and syndromes [2,18,19]. If teeth mature earlier, this may affect the timing of regular examinations of teeth, eruption, occlusion, and referral to orthodontic treatment as well as prophylactic efforts in child and adolescent dental care.

Secular trends may be reversible and demonstrate differences in geography and ethnicity [20]. Accordingly, the present study aims to compare the development of DM between Scandinavian children born 1969–1973 and 2005–2010, respectively.

## 2. Material and Methods

### 2.1. Subjects

In the present study, two groups of ethnic Scandinavian children matched by age and sex were used for the analysis.

**Figure 1:**
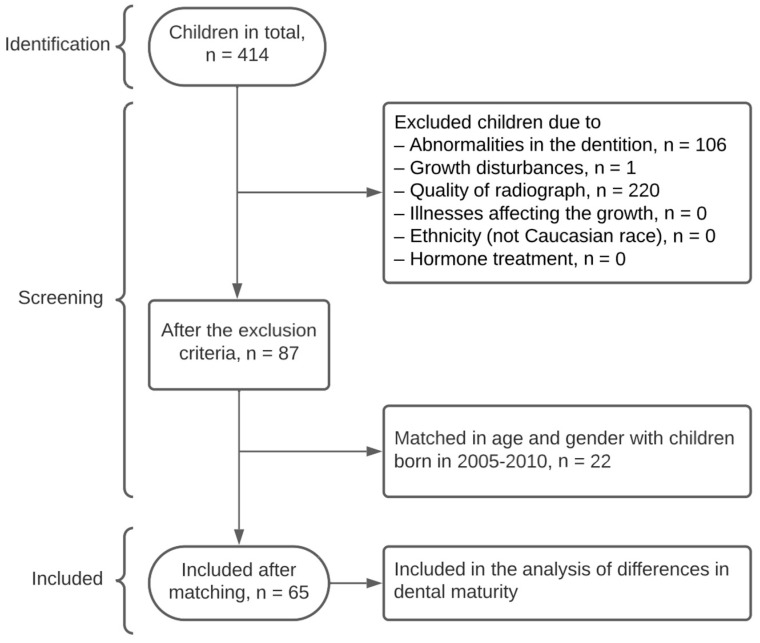
Flowchart of the included children born 1969–1973.

One group consisted of 65 children; 30 girls (ages 9.13 years, range 7.18–12.83 years) at orthopantomography (OP) and 35 boys (aged 9.46 years, range 7.92–14.25 years at OP)—collected systematically from 414 children from a research archive at the Department of Odontology, University of Copenhagen, Denmark (UC-ODON), orthodontic section. The children were born in the period 1969–1973 and received orthodontic treatment at Farum Municipal Dental Health Service, Denmark (Figure 1). The OPs were obtained in the period 1976–1987 and all analysed OPs were taken prior to orthodontic treatment as part of treatment planning.

**Figure 2:**
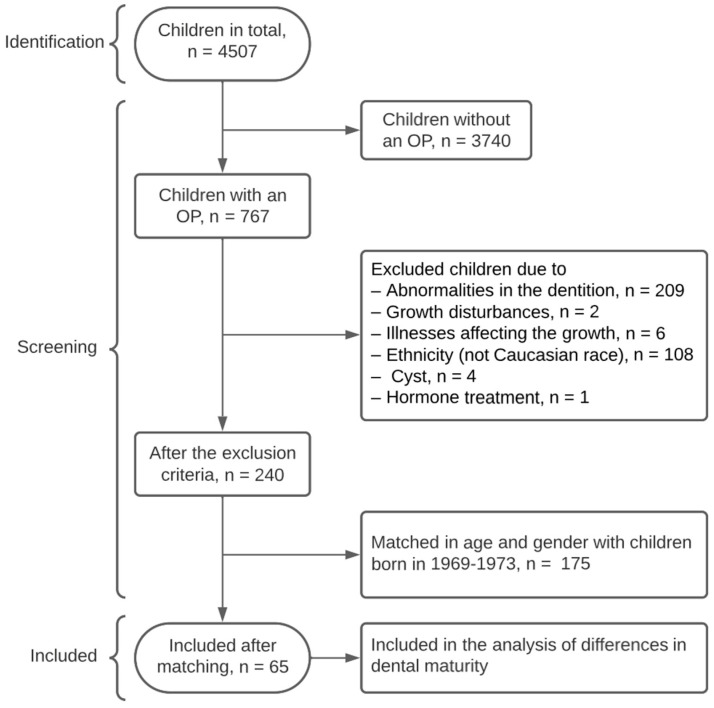
Flowchart of the included children born 2005–2010.

The other group consisted of 65 children; 30 girls (aged 9.17 years, range 7.18–12.83 years at OP) and 35 boys (aged 9.52 years, range 7.92–14.08 years at OP)—collected systematically from 4.507 children born in the years 2005–2010. The selected group of children received orthodontic treatment at the Postgraduate Education of Orthodontics, UC-ODON, or regular dental checks/treatment at UC-ODON (Figure 2). The OPs from the 2005–2010 group were taken in the period 2013–2020. All OPs were taken prior to orthodontic treatment.

The inclusion criteria of the study were: children aged 7–15 years, healthy, and with Scandinavian ethnicity [21,22].

The exclusion criteria of the study were: children with abnormalities in dentition, crowding, growth anomalies, receiving/having recieved hormone treatment, and insufficient quality of the radiograph [21,22] (Figure 1 and Figure 2). Dentition abnormalities included (1) deviations in the number of teeth such as agenesis and supernumerary teeth, (2) malformed teeth in regard to tooth size and tooth morphology, (3) eruption anomalies such as retained teeth.

The power calculation performed prior to the present study was based on previous studies with a similar design. Those studies found a difference in dental maturation of 7.5–10 months among Caucasian children over a period of 30 years [3,9]. In the present study, a difference in the dental maturation of 3.5 months over a period of approximately 37 years is assumed. With a risk of type 1 error of 5% and a type 2 of 20% and with an 80% chance of showing a difference between the two groups, the power calculation shows that approximately 21 children should be present in each group. The ethical standards of the 1975 Helsinki declaration and subsequent revisions were followed. The X-rays were taken by a clinical team as a part of the treatment and followed the guidelines made by the Danish Ministry of Health. Before taking the X-rays, informed consent was given. The Danish Data Protection Agency approved the protocol (J.No: 514-0548/20-3000).

### 2.2. Methods

DM was analysed in children born in 1969–1973 and 2005–2010 by one examiner (ARP). Before analysing the X-rays, the examiner were trained and calibrated by an experienced examiner (LS).

#### 2.2.1. Dental Maturation

DM was analysed from OPs in accordance with Demirjian and Haavikko to illuminate maturity through different methods [21,22]. The OPs of the 1969–1973 group were taken with a Planmeca ProMax 2002 CC panorama X-ray system in the Farum Municipal Dental Health Service, Denmark. The OPs of the 2005–2010 group were taken with Planmeca ProMax 3D Max. The radiographs from 1969–1973 are analogue prints that were analysed on a light board in a dark room. The radiographs from the period 2005–2010 were digitized (ArionG4, Pro Curis AB, Lund, Sweden) and were analysed on a digital display. The examiner was blinded regarding the children’s sex and age.

#### 2.2.2. Demirjian

In accordance with Demirjian, DM stage was assessed on the seven permanent teeth in the left side of the mandibles, excluding the 3rd molar [21]. Each tooth was given a value representing a maturation stage according to eight maturation stages (A–H):

A: calcified dots in the upper part without fusion between them;

B: calcified dots have merged and form cusps;

C: enamel formation of the occlusal surface is complete and dentin formation has begun;

D: crown formation until the cemento–enamel junction is complete and root formation has begun;

E: further root formation, but the root is still shorter than the crown. In molars the initial radicular bifurcation is formed;

F: the root is the same length or greater than the crown;

G: the root formation is complete, but the apex is not closed (distal root in molars);

H: the apex is closed.

Dental age was then calculated according to standard tables for girls and boys and added to a total value, representing the dental age. The DM stages were converted to linear values ranging 1–8 due to the requirements for linear regression analysis [23].

#### 2.2.3. Haavikko

In accordance with Haavikko, the DM stage included the permanent dentition of the maxilla and mandible, including the 3rd molar [22]. The teeth were divided into six maturities describing the formation of the crown and six maturities describing the formation of the root [22]:

O: the crypt is formed, and no calcification is visible;

C_i_: calcified dots in the upper part;

C_CO_: calcified dots have merged and form cusps;

Cr_1/2_: the crown half-formed;

Cr_3/4_: the crown 3/4 formed;

Cr_C_: the crown formation complete;

R_i_: root formation has begun;

R_1/4_: root 1/4 formed;

R_1/2_: root 1/2 formed;

R_3/4_: root 3/4 formed;

R_c_: root formation complete;

A_c_: the apex closed.

All teeth were given a DM stage. Each DM stage was represented by a numerical value from 1 to 12 expressing the twelve maturation stages (O–A_C_) [22].

### 2.3. Statistical Analysis

The statistical analysis was calculated using SPSS version 28.0 (IBM, Inc., Chicago, IL, USA). The results were significant at *p*-values *p* < 0.05. Data were normally distributed, as assessed by quantile–quantile plots (Q-Q-plot). An independent samples *t*-test was performed to test age differences between the two groups. Fisher’s exact test was used to test sex differences between the two groups. The difference in DM between the two groups was analysed separately for boys and girls by a general linear model adjusted for age.

### 2.4. Reliability

One week after the examination 25 OPs were re-analysed to evaluate the intra-reliability of the DM. Cohen’s kappa [24] showed an almost perfect agreement for the methods of Demirjian, K = 0.96 and Haavikko, K = 0.96. The differences between the two evaluations were never more than one maturation stage. In the cases with non-identical outcomes OPs were evaluated and discussed by both authors until agreement was reached.

## 3. Results

The distribution of the children’s ages is shown in Table 1. The study design aimed for identical groups across the age periods by matching the children in both groups by sex and age. Therefore, no significant difference was found in age and sex between the groups (age: *p* = 0.989, sex: *p* = 1.000).

In accordance with Demirjian, differences in dental age between the two groups are shown in Table 2. The mean dental age was significantly higher among children born 2005–2010 in comparison with children born 1969–1973 (*p* < 0.000), corresponding to 6.04 months. When the sexes were analysed separately, mean dental age was significantly higher among girls born 2005–2010 than girls born 1969–1973 (*p* = 0.009), corresponding to 6.49 months, and significantly higher among boys born 2005–2010 than boys born 1969–1973 (*p* = 0.014), corresponding to 5.64 months.

In accordance with Demirjian, differences between the groups in the DM of each tooth are shown in Table 3. All teeth, except the first incisor (I1), second incisor (I2), and first molar (M1), matured significantly earlier among boys and girls born 2005–2010 than boys and girls born 1969–1973 (girls: *p* = 0.001–*p* = 0.015. boys: *p* < 0.000–*p* = 0.033).

In accordance with Haavikko, differences between the groups in the DM of each tooth in the maxilla and mandible are shown in Table 4. In the maxilla, girls’ canines (C) and 2nd premolar (P2) matured significantly earlier in girls born 2005–2010 compared to girls born 1969–1973 (*p* = 0.004–*p* = 0.012). Differences between I1, I2, P1, M1, M2, and M3 were insignificant. Maxillary C, P1, and P2 matured significantly earlier in boys born 2005–2010 compared to boys born 1969–1973 (*p* < 0.000–*p* = 0.005). However, M1 matured significantly later in boys born 2005–2010 compared to boys born 1969–1973 (*p* = 0.031). No significant differences in I1, I2, M2, and M3 were found.

In accordance with Haavikko, in the mandible there were significant differences in girls’ C, P1, P2, and M2, in that they matured earlier in girls born 2005–2010 than girls born 1969–1973 (*p* = 0.001–*p* = 0.030). No significant differences in I1, I2, M1, and M3 were found. Mandibular C, P1, P2, and M2 matured significantly earlier in boys born 2005–2010 compared to boys born 1969–1973 (*p* = 0.001–*p* = 0.015). No significant differences in I1, I2, M1, and M3 were found.

## 4. Discussion

The present study examined whether teeth mature earlier today than 37 years ago. Previous studies have analysed the same issues for other ethnicities and periods [3,9,10,25,26,27]. But it seems that dental maturation has not previously been investigated for Scandinavian children for the periods of 1969–1973 and 2005–2010 when, for the children born 2005–2010, the contemporary material consisted of OPs from the period 2013–2020. As ethnicity has a major impact on DM, it is relevant to investigate changes in DM in Scandinavian children [28,29]. The samples included in the present study showed significant power according to the power calculation, and the samples were matched by age and sex. This is important as girls mature earlier than boys, and age has an impact on maturation [1,30]. The present study was based mainly on children, before orthodontic treatment, which may not represent the general population. Accordingly, a selection bias in the analysis may be present. To minimize bias, the present study used strict inclusion and exclusion criteria in accordance with previous studies [25,26]. Therefore, it is assumed that the results may represent a general population. The present study did, however, have its limitations, as there were no radiographs available of 13- and 14-year-old girls born 1969–1973, thus decreasing the age range. This may be due to the fact that girls and boys mature at different rates causing the beginning of their orthodontic treatment to start at different ages [1,30]. Furthermore, since the study was restricted to children aged a minimum 7 years, it is possible that no significant difference in the maturity of I1 and M1 were found, as the roots of these teeth may already be rooted or almost rooted [31].

This study solely used standard and well-validated methods with a small variability [6,13], and the kappa found in the present study was high compared to other studies with comparable designs [24,26]. The present study compared analogue X-rays with digital X-rays, which may have influenced the analysis of dental maturity, as digital X-rays tend to reproduce dental and bone structures more sharply and with greater contrast [32,33]. Hence, the analysis of the teeth on the digitized X-rays might give the impression of being more advanced. Analog X-rays were analysed in a dark room on a light board to increase the degree of detail.

The present study found a mean difference in the dental age of 6.04 months during a period of 37 years. This is in agreement with previous studies that have found an acceleration in dental age [3,7,8,9,10,11]. A study based on skeletal remains from British children found a difference of approx. six months in dental age across 200 years [10], while a comparable study based on Thai children [8] found a difference in dental age of almost 1 year across 200 years. A study based on Polish children found a difference of approx. 11 months across 30 years [9], and a Croatian study [11] found a difference of slightly less than 9 months across 30 years. Holtgrave et al. [3] found a difference of 6–8 months across 30 years in European boys, based on Nolla’s method.

The reasons behind the observed acceleration of DM are likely multifactorial. From a societal perspective, possible factors that may influence DM are socio-economic status and living standards [2,18,34,35]. The results from the present study suggest a smaller differences in dental age than some of the previous studies [8,9,11]. Increased living standards tend to lead to better nutritional intake, which can affect the DM positively. Some European countries, including Denmark, have had good socio-economic conditions and high living standards for several years [36,37]. Accordingly, changes in these societies may be smaller than changes in other countries, which might explain the smaller differences presented in this paper [36,37].

On an individual level the increased prevalence of obesity might be an important factor. Multiple studies have found a correlation between obesity, advanced dental maturation, and early puberty [2,5,15,16,17]. Children nowadays spend more time in front of a screen and therefore may be more inactive and may have a higher energy intake than earlier generations which can lead to obesity and earlier puberty [15,16]. Dental maturation is likely correlated and influenced by genetics and hormones, which is why earlier puberty may affect dental maturation [12]. Accordingly, changing consumption habits might be part of the reason behind the observed acceleration of DM. It can be difficult to reconcile that both obesity and improved nutritional intake can lead to accelerated DM. The explanation might be that DM is accelerated once the energy intake exceeds to a certain amount, regardless of the nutritional value.

When assessed in accordance with Demirjian and Haavikko, differences in DM were significant for both the mandible and maxilla for both sexes in the present study. For the maxilla, the C and P2 of both sexes and boys P1 matured significantly earlier. For the mandible, C, P1, P2 and M2 matured significantly earlier in both sexes. This pattern disagrees with Jayamare et al. [26], who found that all maxillary teeth, except the canines, matured significantly earlier, but found no significant difference in the mandible across 20 years. Rousset et al. [27], who studied the time of tooth eruption, found that the maxillary P1 and P2 erupted later while the maxillary M2 erupted earlier over 50 years.

While most previous studies have dealt with DM in general, the present study expands the analysis by investigating DM for individual teeth. The present study found that maxillary and mandibular C, P1, P2, and additionally mandibular M2, matured significantly earlier in both sexes, perhaps since these teeth are formed consecutively around ages 8–12 [31]. Accordingly, the results may suggest that tooth maturation is advanced at the age of 8–12 years. In the present study, maxillary M1 matured significantly later in boys, which disagrees with one study [26] that found maxillary M1 matured earlier in boys. There is no obvious explanation for this discrepancy in the results, but it could be due to M1 being the first permanent tooth to erupt [31], usually around the age of six. Since significant differences were found in teeth erupting around ages 8–12, advanced tooth maturation may not affect younger children aged six. Accordingly, it may be the case that advanced maturation does not appear until the age of 8 years, explaining why the advanced maturation is not present for M1 due to its earlier eruption.

In general, the present study showed the largest difference in DM in the mandible, which may be due to the higher growth intensity in the mandible in comparison with the maxilla [38]. Few studies have been able to differentiate between DM in the maxilla and mandible, since Demirjian’s method is the most common and exclusively based on the mandible [21]. The discrepancy between the results may depend on several factors, e.g., the chosen method, the reliability of the analysis, and the choice of the study group. As DM is affected by multiple factors [2,18,34,35], there may be multiple reasons behind the variations in the studies, e.g., geographical, genetic, and cultural factors.

Together with results from previous studies in various populations from different countries, the results from the present study have potentially widespread applications. Knowing the velocity of dental maturation is essential when estimating dental age in forensic dentistry [24], pedodontics, and for timing of orthodontic treatment.

In forensic dentistry, dental age is used to estimate a chronological age of a person of an unknown age [39]. For instance, the chronological ages of refugees seeking asylum as well as unidentified deceased persons are often estimated by the use of their set of teeth [39,40]. The results of the present study may contribute to such estimates. As the study found an advanced dental age of 6 months during a 40-year period, it is possible that children are estimated to be older than their chronological age. Furthermore, since the present study found slightly smaller estimates of the accelerated dental maturation in comparison to previous studies, it highlights that dental maturation might differ across countries and continents.

Additionally, the results of the present study may prove valuable in pedodontics and for the timing of orthodontic treatment. The acceleration of dental maturation might indicate that practitioners should pay attention to, e.g., ectopic eruption of canines at earlier stages than previously assumed. Moreover, accelerated dental maturation might affect the occurrence of malocclusion or conditions in relation to the craniofacial development, which might be a topic of future research.

## 5. Conclusions

The teeth of children born 2005–2010 matured significantly earlier and their dental age was significantly higher in comparison with children born 1969–1973. The difference in dental maturation was significant on maxillary C, P1, P2, M2, and mandibular C, P1, P2, and M2, which matured earlier in both sexes. Maxillary M1 in boys matured significantly later in boys born 2005–2010 in comparison with boys born 1969–1973. The results may prove valuable in forensic dentistry, pedodontics and for timing of orthodontic treatment.

## Figures and Tables

**Table 1:** Overview of the matched age groups and genders for children born in the period 1969–1973 and children born in the period 2005–2010 for comparison of dental maturity.

Age in Years	Number of Subjects		
	Girls (*n*, (%))	Boys (*n*, (%))	Total (*n*, (%))
**7**	7 (23.33)	1 (2.86)	8 (12.31)
**8**	12 (40)	19 (54.28)	31 (47.69)
**9**	3 (10)	7 (20)	10 (15.38)
**10**	3 (10)	2 (5.71)	5 (7.69)
**11**	3 (10)	1 (2.86)	4 (6.15)
**12**	2 (6.66)	3 (8.57)	5 (7.7)
**13**	0 (0)	1 (2.86)	1 (1.54)
**14**	0 (0)	1 (2.86)	1 (1.54)
**Total**	30 (100)	35 (100)	65 (100)

**Table 2:** The difference in dental age for each age group as assessed according to Demirjian when adjusted for age. Only statistically significant results are shown.

Age	Sex	Diff (yrs)	Mths	Lower CI	Higher CI	*p*	*n*
Total	G + B	0.503	6.04	0.23	0.77	0.000	130
	G	0.541	6.49	0.14	0.94	0.009	60
	B	0.470	5.64	0.10	0.84	0.014	70
11	G	0.934	11.21	0.17	1.70	0.030	6

Diff: difference in dental age measured in years; Mths: difference in dental age measured in months; CI: confidence interval; *p*: probability; *n*: number of children. G: Girls; B: Boys.

**Table 3:** The difference in dental maturation of teeth in the mandible in the two groups assessed by Demirjian when adjusted for age. Only statistically significant results are shown.

Tooth	Sex	Mean	Lower CI	Higher CI	*t*	*p*
Total	G + B	0.211	0.11	0.31	4.10	0.000 ^1^
	G	0.222	0.07	0.37	2.98	0.004 ^1^
	B	0.202	0.06	0.35	3.18	0.007 ^1^
33	G	0.482	0.22	0.75	3.67	0.001 ^1^
	B	0.253	−0.04	0.55	1.72	0.033 ^1^
34	G	0.349	0.12	0.58	2.99	0.004 ^1^
	B	0.338	0.13	0.55	3.23	0.002 ^1^
35	G	0.415	0.18	0.65	3.55	0.001 ^1^
	B	0.285	0.04	0.53	2.32	0.024 ^1^
37	G	0.344	0.07	0.62	2.51	0.015 ^1^
	B	0.449	0.23	0.67	4.03	0.000 ^1^

Mean: mean difference in dental maturation stages; CI: confidence interval; *t*: *t*-value for group; *p*: probability; ^1^: significant difference in age. G: girls; B: boys.

**Table 4:** The difference in the dental maturation of teeth in the maxilla and mandible of the two groups as assessed according to Haavikko when adjusted for age. Only statistically significant results are shown.

Tooth	Sex	Diff	Lower CI	Higher CI	*t*	*p*
Total U	G + B	0.386	0.21	0.56	4.40	0.000 ^1^
	G	0.381	0.11	0.65	2.81	0.007 ^1^
	B	0.391	0.16	0.62	3.36	0.001 ^1^
13/23	G	0.427	0.10	0.76	2.61	0.012 ^1^
	B	0.482	0.15	0.82	2.87	0.005 ^1^
14/24	B	0.652	0.23	1.07	3.10	0.003 ^1^
15/25	G	0.705	0.23	1.18	2.98	0.004 ^1^
	B	0.823	0.43	1.22	4.12	0.000 ^1^
16/26	B	−0.229	−0.44	−0.02	−2.21	0.031 ^1^
Total L	G + B	0.417	0.17	0.67	3.33	0.001 ^1^
	G	0.527	0.05	1.00	2.22	0.030 ^1^
	B	0.322	0.09	0.55	2.79	0.007 ^1^
33/43	G	0.360	0.04	0.68	2.23	0.030 ^1^
	B	0.511	0.17	0.86	2.96	0.004 ^1^
34/44	G	0.598	0.26	0.94	3.55	0.001 ^1^
	B	0.424	0.08	0.77	2.49	0.015 ^1^
35/45	G	0.435	0.07	0.80	2.39	0.020 ^1^
	B	0.538	0.12	0.96	2.56	0.013 ^1^
37/47	G	0.533	0.12	0.94	2.60	0.012 ^1^
	B	0.653	0.26	1.05	3.31	0.001 ^1^

Diff: mean difference in dental maturity; CI: confidence intervals; *t*: *t*-value for group; *p*: probability; U: maxilla; L: mandible; ^1^: significant difference in age. G: girls; B: boys.

## Data Availability

The data presented in this study are available on request from the corresponding author.
